# Rapid response in relapsed follicular lymphoma with massive chylous ascites to anti-CD19 CAR T therapy using Piggy Bac: A case report

**DOI:** 10.3389/fimmu.2022.1007210

**Published:** 2022-12-01

**Authors:** Yan Zhang, Zhicai Lin, Faliang Zhang, Xiuxiu Chen, Yaping Yang, Xin Fu, Zhong Li, Yan Sun, Qijun Qian

**Affiliations:** ^1^ Department of Oncology, Shanghai Mengchao Cancer Hospital, Shanghai University, Shanghai, China; ^2^ Clinical R&D Center, Shanghai Cell Therapy Group Corporation, Shanghai, China; ^3^ Shanghai Cell Therapy Research Institute, Shanghai Cell Therapy Group Corporation, Shanghai, China

**Keywords:** non-viral gene transfer, Piggy Bac, follicular lymphoma, relapsed, CD-19 directed, chimeric antigen receptor T cells

## Abstract

CD19-directed chimeric antigen receptor (CAR) T cell therapy has been shown to achieve a considerably durable response in patients with refractory or relapsed B cell non-Hodgkin lymphomas, as seen from the results of Zuma-1, Zuma-5, and other clinical trials. Most of these CARs were generated by lentivirus or reverse adenovirus. It is rare to see CARs using non-viral vectors, such as Piggy Bac (pb), in treating lymphoma patients with active diseases. Generally, patients with a high tumor burden tend to have a higher rate of severe cytokine release syndrome (CRS) or neurological events as reported in the literature. Patients with symptomatic pleural effusions are excluded from the Zuma-1 trial because of the risk of severe CRS. We report here that a patient with relapsed follicular lymphoma with bulky disease and massive chylous ascites failed several lines of chemotherapy. After infusion of the CD19-directed pbCAR-T cells at 6 × 10^6^ cells/kg, the patient had a rapid response and achieved a nearly complete metabolic remission on day 28. There was only grade 1 CRS, and no neurotoxicity occurred. The CAR-T cells reached a peak level on day 14 and spread into the ascites and expanded for 3 months. This might be the first case reported for pbCAR-T cells to treat relapsed follicular lymphoma directly. The long-term efficacy will be observed, and more patients be tested in the future.

**Clinical Trial Registration:**

https://ClinicalTrials.gov, identifier NCT05472610.

## Introduction

CD19-directed chimeric antigen receptor (CAR) T cell therapy has given rise to a long-term efficacy for relapsed or refractory B cell lymphoma and even a potential cure for patients who have attained complete response (CR) (1–4). Zuma-1 showed that the 5-year overall survival (OS) of Axi-cel for large B-cell lymphoma (LBCL) was 42.6% with more than 5 years of follow-up (5). Moreover, Zuma-5 and other clinical trials showed higher rates of durable responses of CD19-directed CAR T cells for indolent non-Hodgkin lymphomas, such as follicular lymphoma and marginal zone lymphoma (6).

Three approved products of CD19-directed CAR T cells are all manufactured by viral gene transfer (7); however, there have been emerging preclinical and clinical trials using non-viral gene transfer-prepared CAR T cells in the treatment of lymphomas or solid tumors. The advantages of the latter method include lower production cost, well-tolerated toxicities, and higher composition of stem cell memory T cells in the products (8, 9). Transposons, as natural non-viral gene delivery vectors, have three types including Sleeping Beauty (SB), Piggy Bac (pb), and Tol2. The pb system is constituted by the pb transposase and a separate transfer plasmid carrying the desired genetic cargo (CAR construct, for instance). It has a higher transposition activity than SB and a larger cargo size than viral vectors; however, the electroporation process for the delivery of transposon vectors might be toxic to the cells, and the transfection efficiency lower than viral vectors (9). In a preclinical trial, mesothelin-targeting CAR T cells, prepared by pb, exhibited a rapid and robust killing effect against pancreatic cancer cells; in the xenograft mice model, they significantly suppressed tumor growth, causing minimal lesions in major organs (10). In a phase I trial treating nine patients with advanced non-small cell lung cancer using EGFR-specific CAR T cells generated by pb, Zhang et al. only reported grades 1 to 3 fever as observed after CAR T cell infusion, without any other symptoms of serious cytokine release syndrome (CRS) (11).

In the Zuma-1 clinical trial, the reported CRS was 92%, in which grade 3 or worse was 11%, and the ICANS was 67%, with grade 3 or worse being 32% (12). Patients with effusions usually had a higher rate of severe CRS and non-relapse mortality rate (13) and were not eligible for Zuma-1 trial (12). Based on our studies of CAR T cells with Piggy Bac in mesothelin and EGFR (10, 11), we generated an anti-CD19 pbCAR T. After evaluation of CD19 CAR T in terms of antitumor activity and safety, we initiated a clinical POC study that was approved by the Board of Ethics of the Shanghai Mengchao Cancer Hospital. We report here a case of relapsed follicular lymphoma with a high tumor burden, including bulky lymphadenopathies and a large amount of chylous ascites, which did not respond to chemotherapy; an anti-CD19 pbCAR T cell therapy was given. The patient rapidly reached nearly complete metabolic remission (CMR) on day 28 positron emission tomography–computed tomography (PET-CT) evaluation. Only grade 1 CRS occurred, and no neurotoxicity was observed.

## Case presentation

### Medical history

A female patient in her mid-40s was initially diagnosed with follicular lymphoma by laparoscopic biopsy in 2015. At her diagnosis, she had multiple retroperitoneal lymphadenopathies, pleural effusion, and ascites. She was given chemotherapy for a total of 12 cycles and achieved remission. She felt well until October 2021, when she palpated an abdominal soft tissue mass on the right lower quadrant. The enhanced CT scan displayed multiple masses in the liver and retroperitoneal lymphadenopathies in the abdominal–pelvic cavity. A laparoscopic biopsy again confirmed a pathological diagnosis with relapsed follicular lymphoma (grade 1, 60%; grade 2, 40%). She received “R-COPP” regimen at a local hospital; however, her ascites progressed. The PET-CT scan showed multiple loci infiltration, including groups of deep and superficial lymphadenopathies, in the liver, bilateral lungs, abdominal–pelvic omentum, and mesentery, right adnexa with 5-cm mass, multiple loci of bone lesions, and a large amount of ascites with medium volume of left pleural effusion. She received two cycles of “R-Gemox” chemotherapy without any response. A pretreatment evaluation confirmed the patient’s high tumor burden with stage IV disease and a follicular lymphoma international prognostic index (FLIPI) score of three points.

The patient met the inclusion criteria, including relapsed follicular lymphoma, no response after two lines of chemotherapy, multiple loci of infiltration, and positive immunohistochemical staining of CD19. She signed the informed consent to be enrolled in the CD19-directed CAR T cell clinical trial.

### Preparation of CD19-directed pbCAR T cells

To manufacture CD19-directed CAR T cells, the patient underwent leukapheresis to collect peripheral blood mononuclear cells (PBMCS). CAR-T cell electroporation, activation, and expansion were performed in the laboratory of the Cell Drug Unit, Shanghai Cell Therapy Group Corporation, as described previously (14). Briefly, autologous PBMCS were electroporated with Piggy Bac transposon and transposase plasmids encoding CD19-directed CAR with a 4-1BB costimulatory and a CD3ζ signaling domain.

### Clinical results

#### Response to CD19-directed pbCAR T therapy

This study was an investigator-initiated trial sponsored by Shanghai Cell Therapy Group Corporation and approved by the Institutional Review Board of Shanghai Mengchao Cancer Hospital. The patient received a lymphodepleting regimen, cyclophosphamide—400 mg (300mg/m^2^) and fludarabine—40 mg (30mg/m^2^), daily on days -5 to -3. Two days later, the patient received a dose of 6 × 10^6^/kg CD19-directed pbCAR T cells infused intravenously.

**Figure 1:**
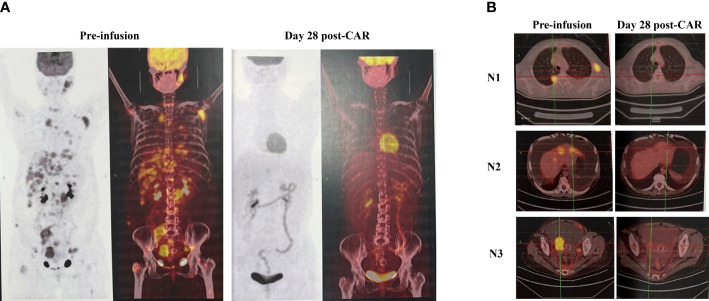
Clinical evaluation by PET-CT imaging. **(A)** Compared with the image before CAR T, PET-CT evaluation on day 28 showed nearly CMR with a few loci in the liver left. **(B)** The lymphoma activities were inhibited after CAR T cell infusion, as seen by PET-CT images for nodules at the lung, liver, and right adnexa. CMR, complete metabolic remission; N, nodule.

She had a rapid shrinkage of her left cervical, axillary lymph nodes, and soft tissue mass located on the right lower quadrant of the abdomen. Her left cervical lymph nodes (30 × 18 mm before infusion) were unpalpated within 1 week and undetected on day 14 by ultrasonography. She had a repeated low-grade fever in the first week after infusion, with the highest temperature of 38.2°C on day 1. She also complained of pain in the waist and bilateral inguinal regions, which was tolerable. She had grade 1 CRS, and there was no evidence of neurotoxicity. On day 28, the PET-CT image showed nearly CMR with a few loci in the liver left with a standard uptake value (SUVmax) of 4.12. However, the adnexa infiltration still existed, and the size decreased from 5 to 2.6 cm, with an SUV of 2.90, similar to that of the liver blood pool (SUVmax = 2.65) (**Figures 1A, B**). At her 3-month re-evaluation by PET-CT, the sizes of the adnexa mass and the liver lesions further decreased, with no uptake of adnexa mass and with one liver lesion left with a little higher SUV. The chylous ascites also gradually decreased, and the drainage catheter was removed on day 43. As of July 24, 2022, the patient was still at follow-up. The main clinical events are outlined in **Table 1**.

**Table 1:** The whole treatment process of the case with relapsed follicular lymphoma.

Date	Clinical event	Clinical results
Sept., 2015	Initial diagnosis	
Sept.~May, 2016	Chemotherapy	CR
Oct., 2021	Relapse	
Nov., 2021	“R-COPP” at local hospital	NR
Dec. 2021~Jan.,2022	2 cycles of “R-Gemox”	NR
19th~21st, Feb.,2022	Lymphodepleting regimen	
24th, Feb., 2022	pbCAR T cell infusion	
24th, Mar., 2022	Day 28 evaluation	Nearly CMR
24th, May, 2022	3 month evaluation	Response ongoing
24th, July, 2022	At follow-up	

CR, complete response; NR, no response; CMR, complete metabolic remission.

#### Pharmacokinetics

**Figure 2:**
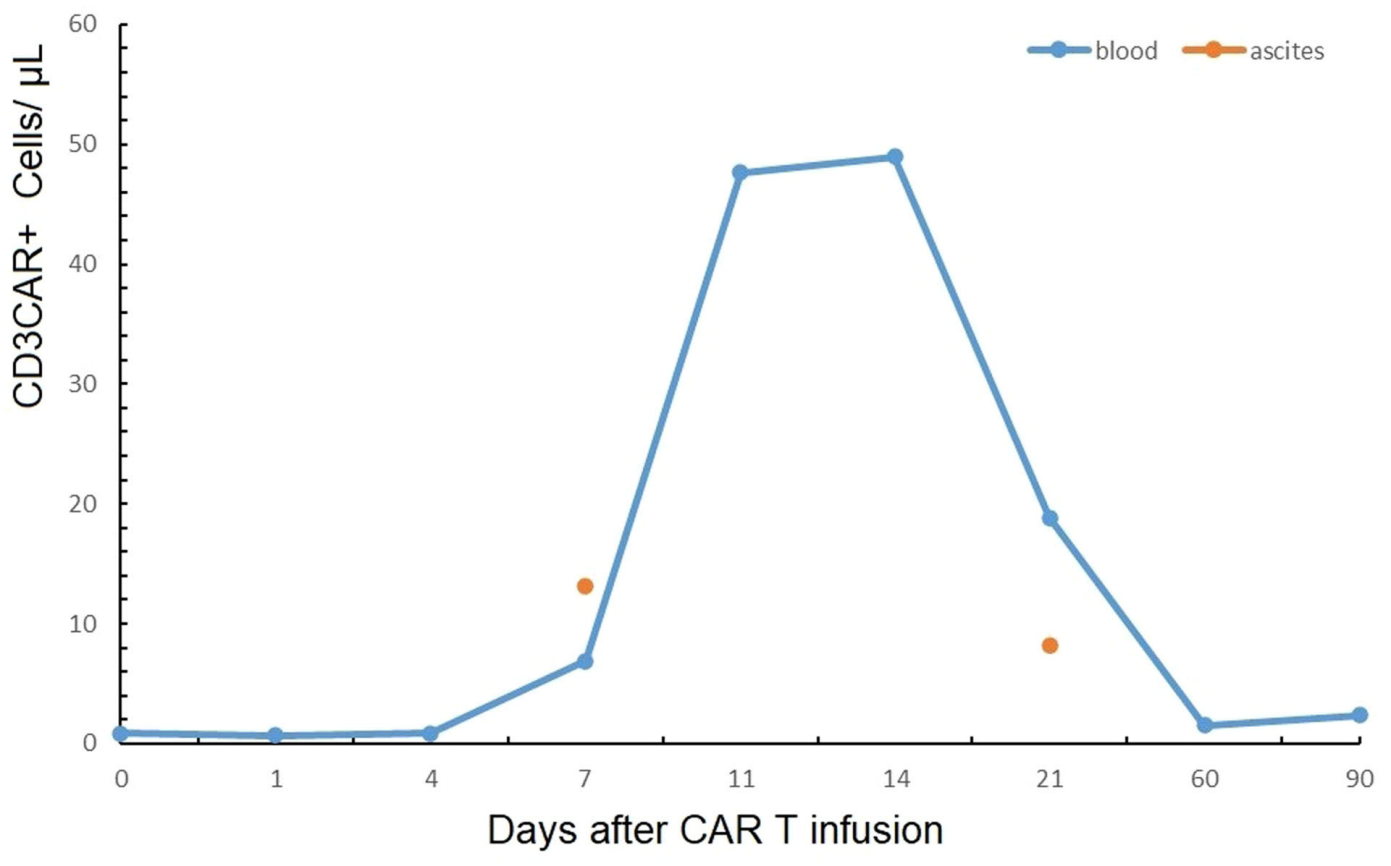
Circulating CAR T cells were detected both in the blood and the chylous ascites by flow cytometry, which peaked on day 14 in the blood.

CD19-directed CAR T cells were detected both in the blood and the chylous ascites by flow cytometry. Circulating CAR T cells were first detected on day 7, and the absolute value was 6.86 μl^-1^. They peaked on day 14, with an absolute value of 48.97 μl^-1^, which accounted for 14.73% of all lymphocytes. Then, the value reduced to 18.85 μl^-1^ on day 21 but maintained detected as 2.38 μl^-1^ at 3 months. The CAR T cells could be detected in the ascites on day 7, with an absolute value of 13.15 μl^-1^, which was higher than that in the blood on the same day. On day 21, the CAR T cells were still detected in the ascites, with a value of 8.13 μl^-1^ (**Figure 2**). The expanded CAR T cells consisted of a certain percentage of immunotypic memory cells, with the CD3^+^CD45RA^-^CCR7^+^CAR^+^cells of 46.68 μl^-1^ on day 11, accounting for most of the CAR^+^ cells, and decreased to 2.88 μl^-1^ on day 21; however, these were still detected at 3 months (**Supplementary Figure S1**).

#### B-cell aplasia, cytokine change, and laboratory testing

The patient had B-cell aplasia before CAR T infusion, which was likely due to a previous Rituximab treatment. The B cell counts remained depleted until day 72, when it recovered to 25 μl^-1^. Repeated intravenous IG was administered when the serum IgG level decreased to lower than 4 to 5 g/L. The level of cytokines detected by flow fluorometry generally showed a fluctuation within the normal range of IL-4, IL-6, IL-10, TNFα, and IFNγ, with a moderate rise of IL-6 on the first day after CAR T cell infusion, which was in accordance with the mild CRS (**Supplementary Figure S2**). The WBCs and neutrophils were reduced sharply after preconditioning and reached the lowest level within the first week after infusion. Granulocyte colony-stimulating factor (G-CSF) was used intermittently. At nearly 2 months, the WBCs recovered to over 3.0 × 10^9^/L. The platelet counts decreased to the lowest level of 91 × 10^9^/L; however, it recovered quickly. The liver enzymes were detected to have slightly increased at 1-month evaluation, probably reflecting the infiltrating CAR T cells that fought against liver lesions.

## Discussion

The three CD19-directed CAR T therapies approved by FDA, Axi-cel, Tisa-cel, and Liso-cel, are all prepared by viral vectors and complex manufacturing processes. The emerging non-viral gene-transferred CD19 CAR T products have not been approved for relapsed/refractory B cell lymphoma. This new approach could potentially reduce the costs and complexity associated with recombinant viral vector-based immunotherapy. There are also other clinical benefits as shown in our case.

In our case, the peak value of pbCAR T cell expansion was 48.97 μl^-1^ on day 14, accounting for 14.73% of all lymphocytes, which was similar to that prepared by viral vectors (15). Interestingly, no severe CRS or neurotoxicity was observed during the pbCAR T treatment, probably because the activated CD19pbCAR-T cells showed relatively low levels of IL-6. Kebriaei P et al. safely conducted a clinical trial using “sleeping beauty (SB)” to generate CD19-specific CAR T cells as adjuvant therapy to treat 26 patients with advanced B cell lymphoma or acute lymphoblastic leukemia following hematopoietic stem cell transplantation (16, 17). Li et al. reported a case of triple-hit relapsed/refractory diffuse LBCL with TP53 mutation, treated with Piggy Bac-generated CAR19-T cells, who obtained a CR in the second month with grade 2 CRS (18). In our case, despite B-cell recovery on day 72 after infusion, the response was still ongoing at 3 months post-infusion, which may be attributed to the pbCAR T cell’s inclination to memory type that kept CAR T cell persistence and the disease under control.

This patient had severe chylous ascites and a medium volume of left pleural effusion before CAR T cell therapy. The patient with pre-CAR T effusions generally had malignant effusions. The fluid accumulations may develop or worsen during the CRS process, leading to significant toxicities and death. In the Zuma-1 trial, patients with pre-existing symptomatic pleural effusions were excluded from the trial. Mirza AS et al. retrospectively analyzed 148 patients receiving CD19 CAR T for LBCL, including 19 patients with a pre-CAR T effusion, and 17 patients without a pre-existing effusion developed a new effusion after CAR T. Compared with patients with no effusions, patients with pre-CAR T effusions had a higher frequency of high-risk baseline characteristics, such as bulky disease and high IPI. Similarly, patients with pre-CAR T effusions had a higher rate of grade 3 or worse CRS (32% *vs*. 5%). Moreover, on multivariate analysis, pre-CAR T effusions were associated with reduced OS and higher non-relapse mortality (13).

The patient in this study had bulky disease with a large volume of ascites before CAR T therapy. She needed drainage every day during lymphodepleting and the first week after CAR T infusion. However, 2 weeks later, we observed a decrease in peritoneal effusions, and the patient needed drainage every 3 or 4 days. The CAR T cells could be detected on day 7 in the ascites, and the absolute value was higher than that in the blood, showing good penetration and infiltration of CAR T cells from peripheral blood to the peritoneum. Lin et al. observed a higher level of distribution of anti-CD19 pbCAR T cells in mesenteric lymph nodes, bone marrow of the femur, spleen, kidneys, and lungs, specifically accumulating at CD19-rich sites and CD19-positive Raji cell-induced tumors (19). During the whole CAR T process, the patient did not feel any pain or discomfort in the abdominal cavity. In the PET-CT on day 28, the left pleural effusion was invisible, and the ascites was remarkably reduced.

The long-term follow-up of Zuma-1 has shown excellent results of Axi-cel for relapsed/refractory LBCL. The overall objective response rate (ORR) was 83%, with a CR rate of 58%, and the 5-year OS was 42.6%. In the trial for relapsed/refractory follicular lymphoma and marginal zone lymphoma, Zuma-5 has reached extraordinary results, which are better than those for aggressive lymphoma. The ORR was 92%, with a CR rate of 76%. With a median follow-up of 17.5 months, the median progression-free survival was not reached. Cappell KM et al. also reported on a long-term follow-up of anti-CD19 CAR T cells for 28 cases with LBCL, eight cases with low-grade B-cell lymphoma, and seven cases with chronic lymphocytic leukemia. The percentage of more than 3-year duration of response was higher for patients with low-grade lymphoma than that for patients with LBCL (63% *vs*. 48%). The median event-free survival (EFS) for all patients with LBCL was 15 months; however, the median EFS for patients with low-grade lymphoma was 55 months (20). So, CD19-directed CAR T cell therapy might be a very powerful therapeutic strategy for relapsed/refractory low-grade lymphoma, with a higher ORR rate and CR rate and good response durability.

Our case of relapsed follicular lymphoma was one with a high tumor burden, involving multiple groups of superficial and deep lymph nodes, liver, lungs, adnexa, omentum, and mesentery. The tumor mass responded rapidly after CD19 CAR T cell infusion, as seen by the unpalpated enlarged cervical lymph nodes within 1 week, and the day 28 PET-CT showed nearly CMR. At 3 months, the response was ongoing. Furthermore, the patient did not have any infection during the whole process, having been given G-CSF, prophylactic antibiotics, and an intermittent supply of immunoglobulin.

In conclusion, Piggy Bac-generated CD19-directed CAR T cells could be a powerful therapeutic choice for refractory or relapsed follicular lymphoma, which showed good efficacy and safety despite the high tumor burden. Long-term efficacy, quality-of-life follow-up, and more cases are warranted for further evaluation.

## Data availability statement

The original contributions presented in the study are included in the article/**Supplementary Material**. Further inquiries can be directed to the corresponding authors.

## Ethics statement

The studies involving human participants were reviewed and approved by the institutional review board (IRB) for Shanghai Mengchao Cancer Hospital. The IRB approval number is SHMCCH-IEC/21-06/05. The patients/participants provided their written informed consent to participate in this study. Written informed consent was obtained from the patient for the publication of any identifiable data/information.

## Author contributions

YZ, ZLin, YS, and QQ designed the study. YZ and ZLin wrote the paper. ZLi revised the paper and gave good advice. YZ analyzed the data and made the figures. FZ and XC were physicians in charge of the patient. Acquisition of data was done by YY and XF. All authors contributed to the article and approved the submitted version.

## Funding

The study received funding from Shanghai Cell Therapy Group Corporation (BZ019). All authors declare no other competing interests.

## Acknowledgments

The authors would like to thank the patient and his family for their participation in and support of the study. We would also like to express our appreciation to all the doctors, nurses, and researchers at the hospital, the cell drug business unit, and Baize medical laboratory who assisted during the study. We thank Professor Dong Chen (Division of Hematopathology, Mayo Clinic, Rochester, MN) for his critical review of the paper and important advice both in Science and English.

## Conflict of interest

Authors ZLin, YY, XF, ZLi, YS, and QQ were employed by Shanghai Cell Therapy Group Corporation.

The authors declare that this study received funding from Shanghai Cell Therapy Group Corporation. The funder had the following involvement in the study: the study design, collection of data and the writing of this article.

The remaining authors declare that the research was conducted in the absence of any commercial or financial relationships that could be construed as a potential conflict of interest.

## Publisher’s note

All claims expressed in this article are solely those of the authors and do not necessarily represent those of their affiliated organizations, or those of the publisher, the editors and the reviewers. Any product that may be evaluated in this article, or claim that may be made by its manufacturer, is not guaranteed or endorsed by the publisher.

## Supplementary material

Supplementary Figure 1 Expansion of CD3^+^CD8^+^CAR^+^ and CD3^+^CD45RA^-^CCR7^+^CAR^+^ T cells. The latter of central memory-like cells could still be detected at 3 months.

Supplementary Figure 2 Change of cytokines. The level of cytokines detected by flow fluorometry generally showed fluctuation within the normal range of IL-2, IL-4, IL-6, IL-10, TNFα, and IFNγ.

## Abbreviations

CAR T, chimeric antigen receptor T; pb, Piggy Bac; CRS, cytokine release syndrome; CR, complete response; OS, overall survival; LBCL, large B-cell lymphoma; CMR, complete metabolic remission; PET-CT, positron emission tomography-computed tomography; FLIPI, follicular lymphoma international prognostic index; PBMCS, peripheral blood mononuclear cells; WBCs, white blood cell counts; SUV, standard uptake value; IG, immunoglobulin; G-CSF, granulocyte colony-stimulating factor; ALT, alanine transaminase; AST, aspartate aminotransferase; SB, sleeping beauty; HSCT, hematopoietic stem cell transplantation; ORR, objective response rate; PFS, progression-free survival; EFS, event-free survival.
