# Supplementary figures and images for: Rapid response in relapsed follicular lymphoma with massive chylous ascites to anti-CD19 CAR T therapy using Piggy Bac: A case report
Source: Front Immunol. 2022 Dec 1;13:1007210. doi: 10.3389/fimmu.2022.1007210 (PMC9752063; doi:10.3389/fimmu.2022.1007210)

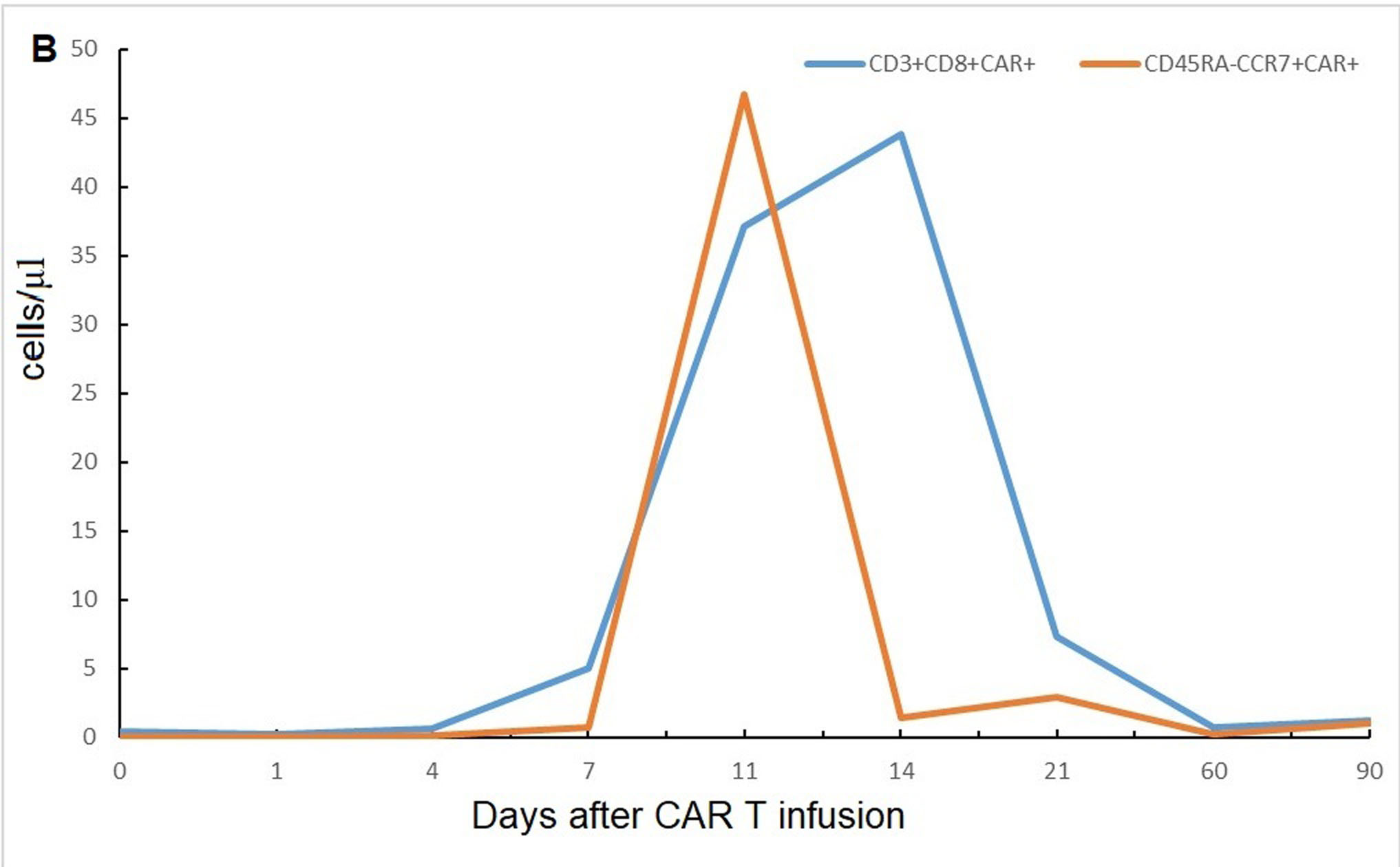

Supplement: Supplementary Figure 1 — Expansion of CD3+CD8+CAR+ and CD3+CD45RA-CCR7+CAR+ T cells. The latter of central memory-like cells could still be detected at 3 months. [file Image_1.jpeg]

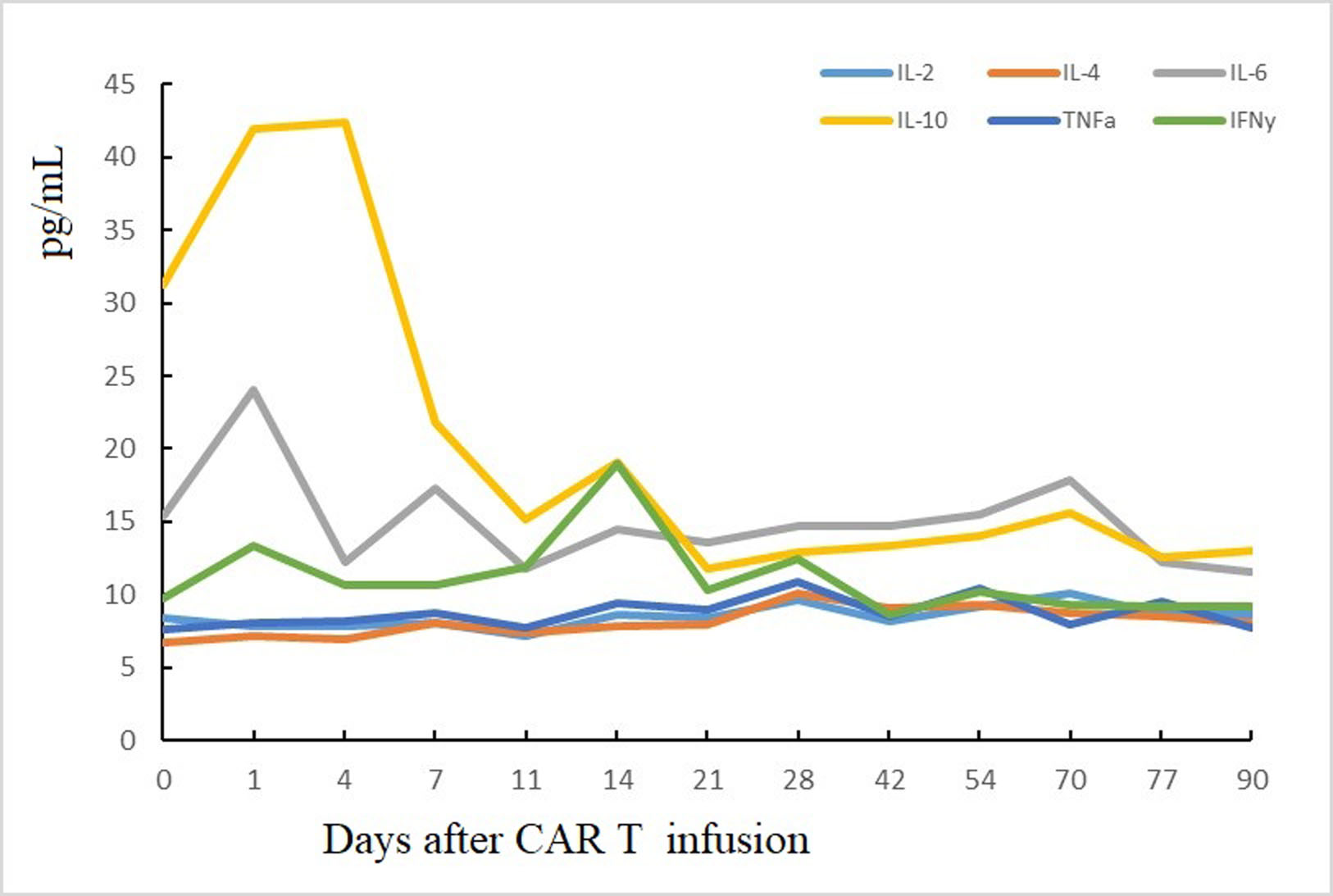

Supplement: Supplementary Figure 2 — Change of cytokines. The level of cytokines detected by flow fluorometry generally showed fluctuation within the normal range of IL-2, IL-4, IL-6, IL-10, TNFα, and IFNγ. [file Image_2.jpeg]
